# Complete Genome Sequence of a *Paenalcaligenes hominis* Strain Isolated from a Paraplegic Patient with Neurogenic Bladder Using Single-Molecule Real-Time Sequencing Technology

**DOI:** 10.1128/genomeA.00252-17

**Published:** 2017-04-27

**Authors:** Rituparna Mukhopadhyay, Joselita Joaquin, Robin Hogue, Austin Kilaru, Guillaume Jospin, Kristin Mars, Jonathan A. Eisen, Vishnu Chaturvedi

**Affiliations:** a Microbial Diseases Laboratory, California Department of Public Health, Richmond, California, USA; b Department of Medicine, Highland Hospital, Oakland, California, USA; c University of California Davis Genome Center, Davis, California, USA; d Pacific Biosciences, Menlo Park, California, USA

## Abstract

The genome of *Paenalcaligenes hominis*, isolated from a paraplegic patient with neurogenic bladder, was sequenced with the Pacific Biosciences RSII platform. The genome size is 2.68 Mb and includes 3,096 annotated coding sequences, including genes associated with quinone cofactors, which play crucial roles in the virulence of Gram-negative bacteria.

## GENOME ANNOUNCEMENT

*Paenalcaligenes hominis* is a Gram-negative, catalase-positive, oxidase-positive, motile, and non-glucose-fermenting rod of the family *Alcaligenaceae*. It was first described in 2010 on the basis of a strain isolated from the blood culture of an 85-year-old man (1). Currently, three species are recognized: *P. hominis* (1), *P. hermetiae* (2), and *P. suwonensis* (3). Due to the lack of whole-genome sequences in the NCBI database from any members of the genus *Paenalcaligenes*, we sequenced a *P. hominis* isolate.

A 23-year-old male paraplegic with neurogenic bladder and indwelling suprapubic catheter presented to the emergency room with flank pain, fever, and no urine output from his catheter for 2 to 3 days prior to admission. A Gram-negative rod grew in pure culture from his urine. The isolate was identified as* Paenalcaligenes hominis*, based on conventional biochemical tests and 16S rRNA gene sequencing. The partial 16S rRNA gene sequence had 99% similarity to that of the type strain of *P. hominis*, CCUG 53761.

DNA was extracted from a culture using the Promega Wizard genomic DNA kit. WGS was performed using the Pacific Biosciences RSII single-molecule real-time (SMRT) sequencing technology. A 17-kb library was prepared according to the manufacturer’s protocol using AMPure PB beads (Pacific Biosciences, Menlo Park, CA). Five micrograms of genomic DNA was sheared using g-Tubes from Covaris (Woburn, MA). The DNA was concentrated, end-repaired, and ligated to hairpin adapters from PacBio. The loading concentration for the library was 0.0125 nM. The template was loaded into SMRT Cell version 3 using a MagBead kit. Sequencing was performed using one SMRT cell, and a 360-min movie was acquired.

The sequence data were assembled using the Hierarchical Genome Assembly Process 3.0 (HGAP 3.0) in SMRT Portal version 2.3.0. After quality filtering and trimming, 232,205 raw subreads were generated, with an average length 5,677 bp, totaling 1,318,310,113 bp. The genome was assembled into one contig of length 2,688,496 bp, with an average coverage of 439.32×. The G+C content of the genome was 48.4%. In order to confirm the identity of the *P. hominis* sequence from the PacBio sequencer, BLASTn was performed. BLASTn of the assembled 16S sequence from *P. hominis* against the NCBI reference RNA sequences (refseq_rna) database aligned 98% to *P. hominis* strain CCUG 53761, with a nucleotide identity of 99%. The neighbor-joining phylogenetic tree analysis of 16S sequence showed that *P. hominis* isolate forms a robust cluster with *P. hominis* strain CCUG 53761 (https://figshare.com/s/41252896e43f1602031f). The PHAST server predicted two prophage regions, with sizes of 22.2 kb and 46.1 kb (4).

Gene predictions and annotations were performed with Rapid Annotations using Subsystems Technology (RAST) database (5–7) and Prokka version 1.1 (8). The annotation for the *P. hominis* genome using RAST showed 401 subsystems, 3,096 coding sequences, and 56 RNA genes (https://figshare.com/s/41252896e43f1602031f). There were 3,093 genes, six rRNAs, 49 tRNAs, and one transfer-messenger RNA (tmRNA) annotated by Prokka. Genes associated with quinone cofactors were found in the *P. hominis* isolate, which play crucial roles in the virulence of Gram-negative bacteria (9).

### Accession number(s).

This whole-genome shotgun project has been deposited at DDBJ/ENA/GenBank under the accession no. CP019697.
